# Assessment of Urinary Osteopontin in Association with Podocyte for Early Predication of Nephropathy in Diabetic Patients

**DOI:** 10.1155/2014/493736

**Published:** 2014-05-04

**Authors:** Abdulrahman L. Al-Malki

**Affiliations:** Biochemistry Department, Faculty of Science, King Abdulaziz University, P.O. Box 80203, Jeddah 21589, Saudi Arabia

## Abstract

*Objectives*. Microalbuminuria has been clinically used for noninvasive evaluation of renal dysfunctions. However, it is a nonspecific marker of diabetic nephropathy (DN). *Methods*. This study was conducted from March 2012 to April 2013 at Biochemistry Unit, King Fahd Medical Research Center (KFMRC). In this study, urinary osteopontin, podocytes number, and levels of immunoglobulin M (IgM) were determined in 60 patients (diabetic normoalbuminuria, diabetic microalbuminuria, and nephritic syndrome) compared with healthy subjects. *Results*. It was found that in diabetic microalbuminuria patients have a highly significant increase in urinary IgM, osteopontin, and podocyte levels as compared to other groups. Nephrotic syndrome patients showed a moderate significant elevation of these parameters compared to control subjects. At a given specificity of 97%, podocytes yielded the highest sensitivity of all markers, 95.5%. The sensitivity was considerably higher compared to IgM and osteopontin. Podocyte number was positively correlated with serum IgM and osteopontin (*r* = 0.63 and 0.56), respectively. Its cutoff corresponding to the 10% coefficient of variation was most appropriate for early diagnosis of DN. *Conclusion*. Monitoring urinary podocyte may provide a noninvasive tool that is a sensitive, accurate, and specific biomarker of glomerular injury and can be used in combination with osteopontin and IgM to more reliably detect and monitor prognosis.

## 1. Introduction

Diabetes mellitus (DM) is considered one of the most chronic diseases affecting people worldwide and the incidence rises to 500 million by 2030 [1]. The diabetic complications including neuropathy, cardiovascular diseases, and nephropathy which is one of the most complications developed in late stage increase the morbidity and mortality. Diabetic nephropathy occurs in approximately 30% of diabetic patients and leads to renal failure in most countries. The first sign of nephropathy was detected by microalbuminuria (presence of albumin in urine) but doesn't cover all patients with renal impairment [2].

Microalbuminuria was detected in patients of severe kidney impairment as hypertension and glomerular basement impairment. Prediabetic hypertensive patients may develop nephropathy in noninsulin-dependent diabetic subjects [3]. Glycemic control is the most important for glucose level in diabetes and the most important is glycated hemoglobin (HA_1_C) as a good index of glucose level [4].

Once diabetic nephropathy was well detected according to the therapeutic protocols, including glycemic and blood pressure, controls were followed up. Diabetic nephropathy can be prevented to a significant degree by early detection using novel biomarkers in body fluids [5].

Podocytes are cells present outside the basement membrane of nephron. In pathological cases, podocytes are expected to be easily found in urine of diabetic patients with micro and macroalbuminuria [6]. Human podocytes have been found to be damaged in diabetic nephropathy patients [7]. An elevation in foot-process pores has been detected in diabetic patients and correlated directly with microalbuminuria [8]. A decreased number of podocytes (podocytopenia) in diabetic patients were detected [9, 10].

Urinary excretion of immunoglobulin M (IgM) was found to be a better index of impaired kidney function than albuminuria in diabetic patients. However, it has not been regarded as an early biomarker of end stage renal disease [11], while plasma cystatin C levels could be a useful marker for renal dysfunction in diabetic patients with normoalbuminuria [12].

Osteopontin (OPN) is a calcium binding protein which is expressed in bone, endothelial cells, and glomerular basement membrane [12]. It is involved in bone turnover and inflammation. In diabetic patients, plasma osteopontin level was significantly correlated with the degree of diabetic nephropathy [13]. In urine, increased OPN excretion level was detected in animal model of acute renal disease [14].

This study was designed for evaluation of urinary podocyte in combination with osteopontin and plasma cystatin C to explore the early, more specific, and sensitive biomarkers for nephropathy in diabetic patients. This is to help the physicians in controlling the occurrence of renal failure.

## 2. Subjects and Methods

### 2.1. Subjects

This study was conducted from March 2012 to April 2013 at the Biochemistry Unit, King Fahd Medical Research Center (KFMRC). Informed consent was obtained from patients and healthy subjects before starting the experiment. The study was approved by the Ethics Committee of King Abdulaziz University Hospital.

This study was conducted on 60 patients (mean age: 37 ± 7 years; 40 males and 20 females) and 20 age-and-gender matched healthy subjects (mean age: 40 ± 8 years; 12 males and 8 females) with normal GFR cutoff value ≥ 90. Patients were admitted to outpatient's King Abdulaziz University Hospital, King Abdulaziz University. The healthy subjects did not have any systemic diseases as diabetes, hypertension, cardiovascular disease, or renal insufficiency.

The patients were divided into three groups as follows. In Group I, the diabetic patients for 10 years were not suffering from nephropathy (normoalbuminuria). In Group II, the diabetic patients for 15 years were with nephropathy (microalbuminuria urinary albumin excretion (20–200 ug/min)). In Group III, there were nondiabetic nephrotic syndrome patients (microalbuminuria).

Blood samples were collected after an overnight fast. Plasma samples were separated and stored at −80°C until assay. Urine samples were collected from all subjects in the morning and were stored at −80°C within 2 h of collection until analysis.

### 2.2. Methods

Plasma samples were subjected to analysis of glucose, glycated hemoglobin, and creatinine levels by spectrophotometer method using kit (Bioline, England). Plasma cystatin C level was measured by the latex agglutination test (ALPCO Diagnostics) assay. In addition, urine samples were subjected to analysis of immunoglobulin M and osteopontin by ELISA kit from ALPCO Diagnostics (Catalog number 41-OPNHU-E01) and detecting podocyte by immunofluorescence using monoclonal antibody against podocalyxin on the surface of podocytes (Table 2). After centrifugation, urine sediment was incubated with anti-human mono podocalyxin antibody (Bioline, England).

### 2.3. Statistical Analysis

Results were expressed as mean ± SD. Sensitivity was calculated as follows: patients with positive markers/all patients; specificity was calculated as follows: patients with negative markers/all patients. Results were compared using Student's *t*-test for continuous variables and chi-square analysis. A *P*  value ≤ 0.05 was considered significant. One-way ANOVA was additionally used as a confirmatory test, complemented by Tukey's test to discover significant intergroup differences. Wilcoxon test was also applied as a nonparametric significance test. ROC curve analysis was used to calculate the diagnostic accuracy of each marker, the “best cutoff” value, and the corresponding clinical sensitivity and specificity.

## 3. Results

Table 1 showed the results of plasma glucose, glycated hemoglobin, creatinine, and the cystatin C levels. There was a significant elevation in plasma glucose and glycated hemoglobin in diabetic normo- and microalbuminuria as compared with nephrotic syndrome and control groups (*P* < 0.001) for both. Plasma creatinine and cystatin C were statistically significantly elevated in diabetic microalbuminuria and nephrotic syndrome patients compared with diabetic normoalbuminuria and control groups (*P* < 0.001) for both. ANOVA analysis showed nonsignificant changes that were observed between nephrotic syndrome and diabetic microalbuminuria. Plasma cystatin C level showed a significant increase in albuminuric level (*P* < 0.001). The level of plasma cystatin C positive correlated with increase in microalbuminuria (normoalbuminuria versus microalbuminuria; *P* < 0.01).

Table 2 referred that urinary IgM was increased about 15-fold in microalbuminuric diabetic patients and nephrotic syndrome patients and compared with control group and normoalbuminuric group. However, osteopontin was elevated about 3-fold in diabetic microalbuminuric group and 1.5-fold in nephrotic syndrome group compared with other groups.

The number of urinary podocytes was significantly elevated in diabetic patients with microalbuminuria and nephrotic syndrome as compared with the diabetic normoalbuminuria (*P* < 0.001). The AUC increased for selected cut off points for podocytes with sensitivity 95.5% and specificity 97%. Positive correlations were observed between urinary podocytes and urinary osteopontin (*r* = 0.56) and IgM (*r* = 0.63), while urinary podocytes and urinary osteopontin were not correlated with other laboratory markers such as plasma glucose, creatinine, and HA_1_C. In multiple regression analysis, plasma cystatin C was affected by creatinine and podocyturia.

Receiver operating curve (ROC) analysis was performed to define the diagnostic profile of urine level markers among subjects with diabetes. The serum level of IgM showed an area under curve (AUC) of 0.90 with sensitivity of 89.0% and specificity of 90%. Also, the urine level of osteopontin supported the diagnostic profile, showing an AUC of 0.73 with sensitivity of 92.3% and specificity of 89,9%. In addition, the urine number of podocytes supported the diagnostic profile, showing an AUC of 0.92 with a cutoff value of 10% (sensitivity of 95.5%; specificity of 97%) (Table 3).

## 4. Discussion

Diabetic nephropathy (DN) is the most common complication of diabetes and leads to renal failure. Here, we will try to find a sensitive biomarker for diagnosis of early phase of diabetic nephropathy. Microalbuminuria is a result of impairment of the filtration of the glomerular basement membrane in diabetes and is used as a predictor of DN. Early diagnosis of nephropathy in diabetic patients is necessary to initiate appropriate treatment.

Cystatin C is a protease inhibitor freely filtrated by renal glomeruli and used as a good marker of renal failure [15]. Cystatin C is produced at a constant rate and released into bloodstream. Its level is mainly dependent on clearance efficiency of the kidney. Patients were categorized into 3 groups depending on their different degrees of kidney damage (normoalbuminuria, diabetic microalbuminuria, or nephrotic syndrome). Plasma cystatin C level was significantly increased in patients with diabetic microalbuminuria and nephritic syndrome compared with other groups. It was thought that this increment was probably due to the tubular phase before glomerular manifestation. This suggests that the cystatin C levels of plasma were related to tubular impairment and can be an earlier measurable marker of renal involvement before onset of albuminuria. This finding indicated that the cystatin C could be an index reflecting renal tubular epithelial cells.

Elevation of serum creatinine and microalbuminuria were the common markers used in renal impairment diagnosis. Recent studies have indicated that serial changes in cystatin, erythropoietin, and collagen can improve sensitivity for early diagnosis. In the present study, serum creatinine and cystatin and urinary IgM, osteopontin, and podocyte levels were determined and correlated to identify the good index for renal impairment.

This study revealed that plasma cystatin C was elevated in association with diabetic microalbuminuria. However, plasma endogenous creatinine depends on creatinine synthesis, metabolism to creatinine, and tubular clearance [16]. Moreover, several tubular proteins are excreted even before the detection of microalbuminuria and elevation of plasma creatinine [17]. Therefore, other biomarkers for evaluation of renal function have been found, and one of them was cystatin C [18]. It was suggested that cystatin C may be one of the additional tubular markers which represent kidney state of diabetic patients.

Results obtained found that renal function of diabetic patients was inversely associated with urine IgM excretion, which indicated that increased urinary IgM excretion was a better predictor of declining kidney function than albuminuria [19]. This is in agreement with the present study that showed a positive correlation between DN and nephrotic syndrome with albuminuria. Since its excretion in urine is associated with nephrotic syndrome, it is a good predictor which may detect the eventual need for renal function monitoring [20].

In the present study, increased OPN level was detected in urinary diabetic albuminuric patients and nephrotic syndrome compared with control and normoalbuminuric patients. United States Food and Drug Administration (FDA) used this marker in order to improve drug discovery [21]. In this study, diabetic patients with renal impairment showed significantly elevated urinary OPN level compared with other groups [22].

This finding suggested that urinary OPN might act as a predictive diagnostic marker because of its further prognostic value. The trend towards elevated urinary OPN in diabetic subjects resulted from enhanced glomerulus dysfunction. However, elevated urinary OPN level as a result of diabetic renal injury [23] may be important for evaluation of kidney efficiency.

Podocyte impairment was found in renal insufficiency. Podocytes in urine can be found in diabetic patients with micro- and macroalbuminuria [24].

Urinary podocytes were not detected in the normoalbuminuric subjects, 95% at the diabetic microalbuminuric stage and 87% at the nephrotic syndrome, which indicated that urinary podocytes might be a useful biomarker for detecting early glomerular injury in diabetic patients [25].

Damage to the glomerulus is a good measure of kidney dysfunction. Such damage invariably leads to protein loss, known as proteinuria, and might possibly result in cell sediment shedding. Intermediate-to-high molecular weight proteins such as albumin, if found in urine and not caused by pre- or postrenal factors, are indicative of kidney damage. Additionally, special glomerular cells, known as podocytes, are also thought to be markers of glomerular damage if found in urine. The podocyte layer comprises one of the three main components of the glomerular filtration barrier.

In conclusion, these biomarkers showed good predictive values with regard to ROC-AUC (0.21 and 0.65 for podocyte and osteopontin, resp.) and higher sensitivity and specificity.

## Figures and Tables

**Table 1:** The levels of fasting plasma glucose, glycated hemoglobin, creatinine, and cystatin C in all studied groups (mean ± SD).

Parameters	Patients groups			
	Healthy control *n* = 20	Diabetic (normoalbuminuria) *n* = 20	Diabetic (microalbuminuria) *n* = 20	Nephrotic diseases *n* = 20
Glucose (mg/dL)				
Mean ± SD	90.8 ± 8	140 ± 16	149.7 ± 11	109.3 ± 8.2
*P*_1_ value	—	<0.001	<0.001	N.S
*P*_2_ value	—	—	<0.001	<0.05

Glycated hemoglobin (%)				
Mean ± SD	4.1 ± 0.3	7.5 ± 0.5	9.7 ± 0.8	5.2 ± 0.2
*P*_1_ value	—	<0.001	<0.001	N.S
*P*_2_ value	—	—	<0.001	<0.001

Creatinine (mg/dL)				
Mean ± SD	0.8 ± 0.02	0.9 ± 0.06	1.9 ± 0.09	2.1 ± 0.07
*P*_1_ value	—	N.S	<0.001	<0.001
*P*_2_ value	—	—	<0.001	N.S

Cystatin C (mg/dL)				
Mean ± SD	0.8 ± 0.02	0.9 ± 0.02	1.3 ± 0.03	1.4 ± 0.04
*P*_1_ value	—	N.S	<0.01	<0.001
*P*_2_ value	—	—	<0.001	N.S

*n* = number of cases. *P*_1_ value: diabetic normoalbuminuria and nephritic syndrome versus control. *P*_2_ value: diabetic microalbuminuria versus diabetic normoalbuminuria.

**Table 2:** The levels of urinary immunoglobulin M, osteopontin, and podocyte in all studied groups (mean ± SD).

Parameters	Patients groups			
	Normal control *n* = 20	Diabetic (normoalbuminuria) *n* = 20	Diabetic (microalbuminuria) *n* = 20	Nephrotic diseases *n* = 20
IgM (ng/L)				
Mean ± SD	2.1 ± 0.06	3 ± 0.3	31.7 ± 2.8	26.3 ± 2.2
*P*_1_ value	—	N.S	<0.001	<0.001
*P*_2_ value	—	—	<0.001	N.S

Osteopontin (U/L)				
Mean ± SD	120.8 ± 23	130 ± 56	312.7 ± 25.8	179.3 ± 28.2
*P*_1_ value	—	N.S	<0.001	<0.01
*P*_2_ value	—	—	<0.001	<0.001

Podocyte (cells/mL)				
Mean ± SD	Nil	Nil	3.9 ± 0.07	2.3 ± 0.2
*P*_1_ value	—	—	—	<0.001
*P*_2_ value	—	—	—	<0.01

*n*  = number of cases. *P*_1_ value: diabetic normoalbuminuria and nephritic syndrome versus control. *P*_2_ value: diabetic microalbuminuria versus diabetic normoalbuminuria.

**Table 3:** Receiver operating curve (ROC) analysis of investigated urine parameters as a test for diagnosis of diabetic nephropathy.

Variable	IgM	Osteopontin	Podocyte
AUC	0.9	0.73	0.92
Sensitivity (%)	89%	92.3%	95.5%
Specificity (%)	90.4%	89.9%	97%
